# Jefferson County [Alabama] Medical Society

**Published:** 1891-09

**Authors:** 


### JEFFERSON COUNTY [ALABAMA] MEDICAL SOCIETY.

From the *Alabama Medical and Surgical Age*, August.

The President, Dr. T. L. Robertson, in the chair.

The Secretary being absent, Dr. W. W. Ransom was appointed secretary *pro tempore*.

The regular essayist for the evening was not present, and Dr.

W. E. B. Davis was requested by the Society to report some of his

RECENT WORK IN ABDOMINAL SURGERY.

The following is a synopsis of his report:

Case I.—About the 1st of June he had been requested by Dr. Jolly, Superintendent of the Hospital of United Charities, to examine at the hospital, Betty B., who had been delivered of a healthy child two months previously. Since that time she had suffered continuously, and was seldom clear of fever. Her temperature would often go to 102½ degrees. There was a distinct enlargement of the ovary on the right side—the side on which she complained of so much pain—which was evidently a puerperal septic inflammatory enlargement. Vaginal tampons saturated with equal parts of glycerine and iodine were advised, and also tonics and nutritious diet; and if she did not begin to improve in two or three weeks, it was advised that the diseased tube and ovary be removed. She grew worse, and the latter part of June he was again requested to see her by Dr. Cochran, the attending physician of the hospital, and, after the patient had been anesthetized, the enlarged ovary and tube with extensive adhesions could be readily made out. The patient had already acquired the habit of taking opium and was anxious for an operation in order to be relieved of her suffering. On Friday, the 10th of July, in the presence of Drs. J. D. S. Davis, Jolly, Cochran, Perry, Wm. Luckie, and others, he made a section and found very extensive adhesions, and an ovary the size of a large walnut, and a very large occluded tube. There was but little trouble in the left ovary or tube, but the organs on both sides were removed, and the fundus of the uterus attached to the anterior wall of the abdomen. The cavity was flushed and a glass tube introduced. The operation lasted about thirty minutes, and the patient progressed as well as could be expected in a morphine case. There was a mural abscess which gave but little trouble. She is now sitting up, and does not take morphine—does not even want it. It may be of interest to say that her temperature went up twice to 104 degrees, merely from nervous excitement. It only remained up a very short while each time.

Case II.—Elsie B., aged 27; colored. This woman had been operated on four months previously, and a cyst of the right ovary removed. After she had gotten up from the operation, there began to develop a hard tumor above the umbilicus, with a temperature of 102 degrees in the afternoon. She had but little appetite and did not digest what she ate. She did not improve, but continued to grow worse, as the tumor grew large. It was explained to her husband that this was most probably a malignant growth, but that it might be an abscess. If the latter, there was hope of relieving her by an operation, but if malignant, the operation could not benefit her. She was advised to go into the hospital for an exploratory incision, which she did, and the operation was done on Friday, July 1st, in the presence of Drs. J. D. S. Davis, and Wm. Luckie, and Mr. Williams, and others. The incision was just long enough to explore with two fingers, and there was no difficulty in making out a cancer of the mesentery. The operation lasted only twelve and a half minutes, and the patient never seemed to have any symptoms from it. The incision has healed perfectly without pus, but the patient is gradually dying from the cancer.

CASE III.—On the same day that he operated on Case II. he was called to Pratt Mines by Drs. Brown and Cunningham to see J. Kellar, aged 19 years, who had been shot with a 42-calibre pistol at 11 o'clock the night before. The ball entered one and a half inches from the spinal column, and just below the last rib on the left side. A probe would enter the cavity through the opening. There was blood in the urine, and the abdomen was very rigid, giving evidence of a general peritonitis. His temperature was 100½ degrees and pulse 100. It was decided to have him brought to the hospital in Birmingham, a distance of five miles, to be operated on. At 3.30 P. M., seventeen and a half hours after the injury, an exploratory section was made in the presence of Drs. Geo. Brown, Cunningham, J. D. S. Davis and others. The incision was four inches long, external to the left rectus muscle, with its center a little above the umbilicus. The abdomen contained a large quantity of dark, dirty-looking blood, and there was present a diffused peritonitis. The ball had passed through the kidney and spleen, but had not injured the intestine. It was removed from the internal anterior wall of the abdomen, where it was lodged, which was on a direct line with the wound of entrance. After washing out the cavity thoroughly and replacing the intestines, which was done with some difficulty, owing to distension—all bleeding being stopped—the incision was closed and a glass drainage-tube placed in the lower angle of the wound. The operation lasted thirty-five minutes, and the patient did not suffer shock. He lived forty hours, and died from the general septic peritonitis, which existed at the time of the operation. It was impossible to get the bowels to act, owing to the extreme paralysis from the inflammation. The peritonitis was evidently caused by the urine in the blood, which escaped from the kidney, and proves the necessity of prompt operative procedure, not only in cases where the bowel is perforated, but also in kidney injuries, in order to keep the patient from dying from peritonitis. The man could not have been saved by any procedure at the time he was operated on. Just here it should be stated that it would have been better to incise the gut in this case, so that the gas could have escaped, and allowed the bowel to be replaced with less traumatism. The wound of entrance was opened by a free incision and drained.

CASE IV.—On Thursday, July 16th, he removed the ovaries and tubes from Mrs. Ella D., aged twenty-eight, at the hospital, in the presence of Drs. J. D. S. Davis, Chas. Hickman, William Luckie, and others. She had been treated for uterine hemorrhage by the curette electricity, Emmet's operation on the cervix, etc., but without permanent benefit, and the bleeding continued to grow worse, and she continued to lose flesh. There was considerable pain in both ovarian regions, with all the symptoms of salpingitis. The operation lasted eighteen minutes, and the patient recovered without an unfavorable symptom. She is now able to sit up in bed.

Case V.—On the same day that he operated on Case IV., he received a telegram to go to Columbiana "prepared to do laparatomy," and found Mrs. L., aged fifty years, with an umbilical hernia as large as a child's head, which had been strangulated for fifty hours. Her pulse was 130, and feeble. It was decided to give her the benefit of an operation, notwithstanding her extreme condition, Davis's catgut mats and Senn's bone plates were placed in readiness, so that they might be used if the bowel had been destroyed by the strangulation, provided the patient's condition would permit of it. After the incision was made, which was quite free, the bowel was found extensively attached, and there were already two large holes in the intestine, which could not be remedied except by resection and anastomosis. The patient was very feeble, and there was nothing to be done except to make an artificial anus. It was apparent as soon as the sphacelated condition of the bowel was seen, that the operation had been done too late, for the patient's condition was so extreme that she could scarcely have been expected to recover after an operation for the relief of a simple strangulation.

Case VI.—Mrs. M. A. L. had been seen in consultation with Drs. Finch and Barrett, of East Lake. She was thirty years of age, and for two years had suffered with extreme procidentia. She had not been able to keep the uterus in the vagina for some years—not since the birth of her only child, a boy of eight years of age. For several months she had been suffering extreme pain in both ovarian regions, and had been confined to the bed a great deal, and has recently had to take opium constantly. The body of the uterus was bound down, and while the canal measured six inches, it was not prudent to attempt an operation on the cervix. The elongation of the neck was above the vaginal insertion. She was advised to enter the hospital for operation, which she readily assented to; and on Monday, July 20th, she was operated on in the presence of Drs. Finch and Barrett and C. C. Jones, of East Lake, Dr. Jones, of Woodlawn, Drs. J. D. S. Davis, William Luckie, and others. After the incision was made, there was found the most extensive adhesion he had ever seen. Bowels and everything were attached. It was very difficult to make out anything. A hematosalpinx of large size was found on the right side, and an ovarian cyst, as large as a small orange, on the left side. After their thorough removal, the uterus was brought up and attached to the abdominal wall by the two lower stitches, which passed through one side of abdominal wound, then through the wall of fundus, and then through the other side of the abdominal wall. When these two ligatures were tied, they closed the lower angle snugly and brought the uterus up against the abdominal wall. The abdomen was flushed with hot water, and a glass drainage tube put in, which was removed in twenty-four hours. This case did not do well for a while, a large abscess having developed in the abdominal wound. She had a very rapid pulse for three days. Since that time she has been doing well, and now, eight days after the operation, says she feels well enough to be out of bed.

CASE VII. — This is a case in which suprapubic cystotomy was done for the removal of a stone. The patient, a boy eight years of age, was sent to him by Dr. Cotton, of Ensley, and was operated on in the hospital, Saturday, July 25th. He has had no unfavorable symptoms since the operation, which lasted twelve minutes. He thinks the high operation preferable in all conditions requiring the bladder to be opened.

After the discussion of these cases, Dr. J. D. S. DAVIS presented a perforated appendix removed after death from a boy upon whom he had performed two sections for the removal of pus from the abdomen. Dr. Davis first saw the boy, aged twelve years, February 1, 1891. He had been treated by Dr. Cooper for fever, accompanied with occasional attacks of dysentery, when he discovered an enlargement on the right side, extending from the right iliac region to the border of the ribs. When Dr. Davis first saw the patient, his pulse was 120 per minute and temperature 102°. He had frequent actions accompanied with bloody discharges. An immediate operation was advised, but was unavoidably postponed. Fifteen hours later the patient was anesthetized and abdomen opened, by an oblique incision three inches in length, beginning above the spine of the right ilium. The abdomen was found to contain pus from a recently ruptured abscess, and two abscess sacs unruptured. The abscesses were broken up and contents removed, which was about a pint of very offensive pus. The abdominal and abscess cavities were drained by a double rubber and glass drainage-tubes. The abdominal incision was closed by including the peritoneum in the sutures. The drainage tubes were kept dry by a long-nozzled rubber piston syringe. The temperature the first day after the operation was 100°; pulse, 100 ; second day, temperature 98½°; pulse, 105 ; third day, temperature 99°; pulse, 105 ; fourth day, temperature 98½°; pulse, 105. After the sixth day the temperature remained normal, pulse gradually getting stronger. On the third day the glass drainage-tube was removed; the stitches on the sixth; the rubber tube was accidentally displaced and opening completely closed on the ninth day. The patient made a rapid and uninterrupted recovery, and was on the street on the eighteenth day. He enjoyed perfect health until July 8th, when he was seized, while playing ball, with a sharp pain in the abdomen. A half-teaspoonful of paregoric was administered, and Dr. Whelan was summoned. He found the patient easier, with a slight fever and excited pulse. After learning a complete history of the case, he advised an immediate exploratory incision. The mother refused. He was then placed on Epsom salts and the bowels freely purged. Dr. Davis saw the case in consultation July 10th, when all the symptoms were very much relieved; temperature reduced to 100°; pulse, 95; and swelling subdued. Under the favorable improvement, operation was postponed on the ground that if he had suppurative peritonitis the operation at so late a time could not be expected to save him; and, on the other hand, if there existed no rupture or perforation, but simple peritonitis (non-suppurative), the salts so far had met every indication. Dr. Whelan saw the patient every five hours, and on the following day Drs. J. D. S. Davis, W. E. B. Davis and T. L. Robertson were in consultation. Temperature then was 103°; pulse, 125; brow contracted and facial expression anxious. An immediate laparatomy was decided upon, with little hope of recovery. An incision was made in the right iliac region over the appendix, and a large quantity of offensive pus removed. An unsuccessful, hurried search was made for the appendix, the cavity well irrigated, a glass drainage-tube inserted and the incision closed. The patient died forty hours after the operation. The necropsy revealed numerous adhesions, perforated appendix buried beneath the cecum and surrounded by a ruptured tumor wall. The walls of the appendix were very thick and indurated. A radical operation had been advised in this case after recovery from the first.

Birmingham, July 28, 1891.

